# Potential Efficiency of Riparian Vegetated Buffer Strips in Intercepting Soluble Compounds in the Presence of Subsurface Preferential Flows

**DOI:** 10.1371/journal.pone.0131840

**Published:** 2015-07-06

**Authors:** Suzanne Edith Allaire, Claudia Sylvain, Sébastien F. Lange, George Thériault, Pierre Lafrance

**Affiliations:** 1 Département des sols et de génie agroalimentaire, Université Laval, Québec City, Quebec, Canada; 2 Soils and Crops Research and Development Centre, Agriculture and Agri-Food Canada,Québec City, Quebec, Canada; 3 Institut National de la Recherche Scientifique, Centre Eau Terre Environnement, Québec City, Quebec, Canada; Sun Yat-Sen University, CHINA

## Abstract

Buffer strips have been widely recognized as to promote infiltration, deposition and sorption of contaminants for protecting surface water against agricultural contamination. However, such strips do not intercept all contaminants, particularly soluble ones. Although preferential flow (PF) has been suggested as one factor among several decreasing the efficiency of buffer strips, the mechanisms involved are not well understood. This project examines buffer strip efficiency at intercepting solutes when subsurface PF occurs. Two soluble sorbed tracers, FD&C Blue #1 and rhodamine WT, were applied on an agricultural sandy loam soil to evaluate the ability of a naturally vegetated buffer strip to intercept soluble contaminants. Rhodamine was applied about 15 m from the creek, while the Blue was applied 15 m to 165 m from the creek. Tracer concentration was measured over a two-year period in both the creek and the buffer strip through soil and water samples. Although the tracers traveled via different pathways, they both quickly moved toward the creek, passing beneath the buffer strip through the soil matrix. Our results demonstrate that the risk of water contamination by soluble contaminants is high in such systems, even when a well-vegetated buffer strip is used. The design of buffer strips should be modified to account for underground bypass, either by using plants that have deep, fine roots that do not favour PF or by adding a filter extending deep underground that can be regularly changed.

## Introduction

Vegetative buffer strips are considered to be one of the best practices for reducing contaminant transport toward surface water [1–2]. Their efficiency in capturing contaminants at the soil surface, particularly when runoff occurs, is well recognized [3–5]. This is mostly attributed to their ability to intercept water and sediments by decreasing the speed of runoff.

The effectiveness of such barriers is nevertheless highly variable and less known for below ground movement. This variability is explained in part by the multiplicity of processes and factors involved and by their dynamic and complex nature [6]. Width, slope, climate, soil, topography, and hydrological processes, all influence their efficiency [2, 4, 6, 7]. Soil wetness determines the distribution between surface and subsurface flows at the onset of and during rainfall events [8]. At the surface, soil detachment occurs during long or intense rains. Detachment and sedimentation may favour ridges and gullies, concentrating the flow at local outlets and causing surficial preferential flows that either bypass the strips or decrease their efficiency at the soil surface [8–10].

The density of the buffer strip vegetation and its resistance to bending are also determining factors of buffer strip efficiency at the soil surface [8, 9]. However, the efficiency of roots for contaminant extraction is much less known. Contaminant properties also influence buffer strip efficiency [9–11]. Although it is usually assumed that, except for surface runoff, highly sorbed contaminants do not move in soil [6], it have been shown that they may infiltrate belowground through vertical preferential flow (PF) paths such as earthworm burrows and cracks. In addition, the authors of one study observed both a decrease in total phosphorus and an increase in soluble phosphorus in surface water in presence of buffer strips [10]. Soluble contaminants seem more likely to bypass buffer strips than strongly sorbed ones, because the former rely on water movement rather than on particle movement [11]. However, the ability of buffer strips in intercepting soluble contaminants during their migration belowground is not well understood. Soluble contaminants may reach greater depths by moving with the water either through standard advection–dispersion movement in the soil matrix or by vertical PF processes.

Transportation time in the subsurface generally decreases with an increase in slope, in the presence of shallow soil, with low permeable soil underneath the surficial one, with artificial drainage (constructed lateral preferential flows), in the presence of large lateral roots or biopores which creates lateral preferential flows called ‘pipe flows’, with other lateral preferential flow processes, and when there is sufficient precipitation to trigger water movement [12]. Within the subsurface, vertical processes carry contaminants downward [13–16], while horizontal processes transport them toward surface water. The connectivity between vertical and horizontal processes governs the overall reaction time of the field with respect to contaminant transport toward surface water.

Particles may move below ground at high speed when in natural or artificial pipes or in concentrated flow paths [17]. Natural pipes develop along large roots, such as those produced by trees, or along other long, narrow obstacles. Because water detaches soil particles every time it flows through these pipes, they enlarge over time through positive feedback, resulting in faster lateral flows (LF) occurring more frequently at higher flow rates over time [17]. In such cases, contaminants reach the buffer strip below the root zone at high concentration and are therefore less subject to degradation and extraction by plants. These PF paths may continue through the buffer strip, directly reaching surface water. The presence of subsurface PF has sometimes been suggested to explain the poor interception capacity of particular buffer strips [18–22].

These belowground PF processes and bypass systems have been put forward as potential explanations for buffer strip inefficiencies with regard to various soluble agricultural contaminants. To our knowledge, there have been very few field studies that have tested this hypothesis. The goal of this study was to examine the ability of buffer strips to intercept solutes in a loamy soil where subsurface PF is suspected to occur at the field-scale using soluble tracers.

## Materials and Methods

### Site description

The field experiment was conducted on private land in the Bras d’Henri watershed at 46°28'51.5" N and 71°13'03.9" W in Quebec, Canada. The landowner gave his permission for the study on condition that we not modify his field or soil management regime. The study did not involve endangered or protected species.

**Fig 1:**
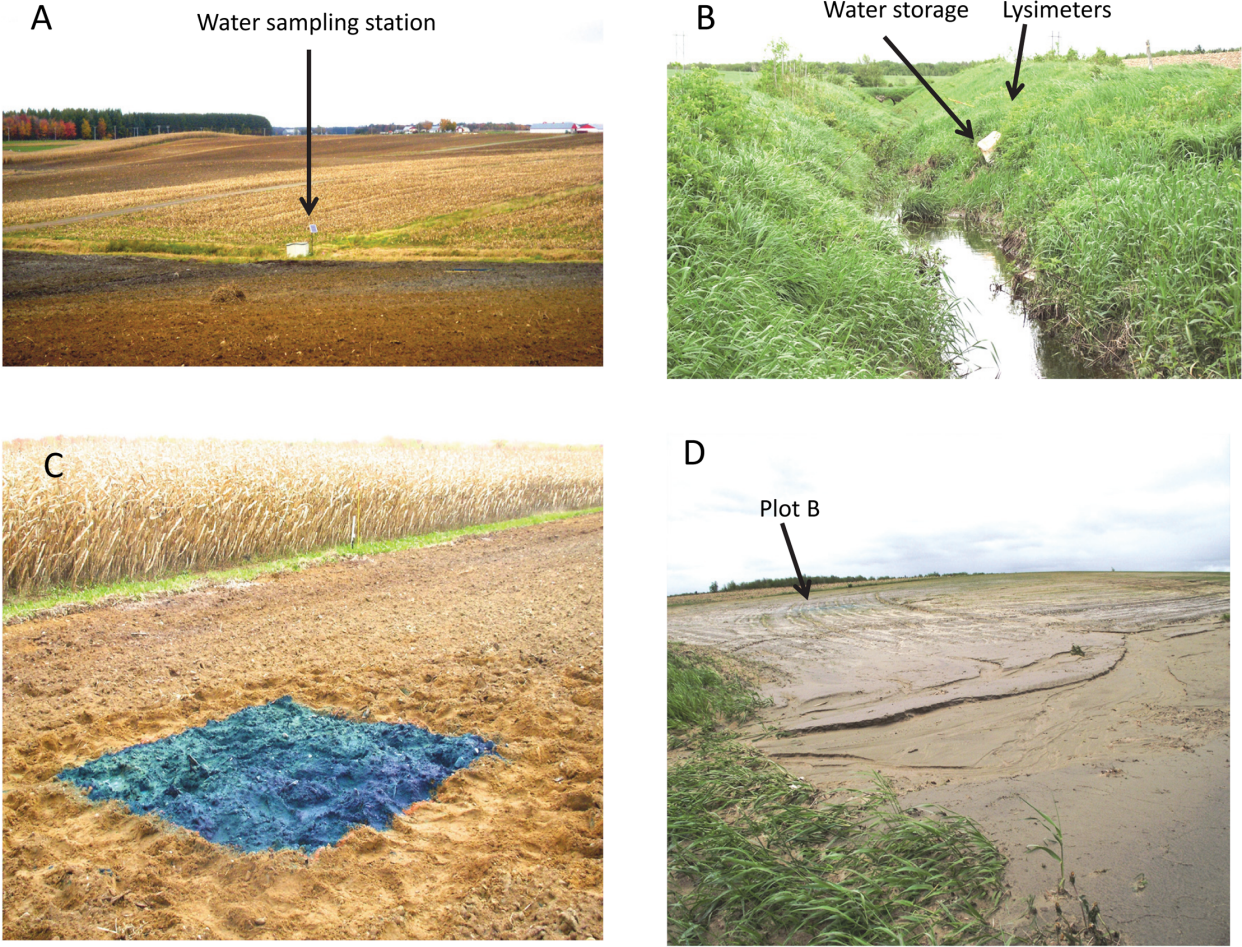
Photos of (A) the general aspect of the field, (B) the buffer strip, (C) Blue and rhodamine application on plot C, and (D) gullies near plot B and interception of particles in the buffer strip grass after the most intense rain during the studied period (Photos: Soil Physics and Hydrodynamic Group, Université Laval).

The field (Fig 1A) is a catena with Beaurivage soils, with a well-developed podzol on the top of the hill, a gleyed brunisol on the backslope, and a poorly drained gleysol at the footslope [23] connecting to a small creek that flows year round. Previously forested, the site was cleared about 20 years ago, at which time a small number of artificial drains were installed and tree stumps were buried at various depths. The soil was cultivated with cereals from 2004 to 2006, corn from 2007 to 2008, soya in 2009 and 2010, oats in 2011, alfalfa in 2012, and then again with corn. The slope of the field is about 4% (Fig 1A).

A naturally riparian vegetated buffer strip 7 m wide was installed in 2004 (Fig 1B). The buffer strip can be subdivided into 2 sections, the creek bank, about 3 m wide, and a relatively flat section about 4 m wide. The soil in the buffer strip (Table 1) was very similar to that of the field (data not shown) but with more gravel and rocks (>5%) of various sizes, particularly in the creek bank section and in the subsurface. The soil is a fine sandy loam slightly finer with depth. The saturated hydraulic conductivity, Ks (Table 1), indicates that water movement is relatively slow in the buffer strip soil, and greater in the horizontal than in the vertical direction. The density of the soil (BD, 1.4 Mg m^-3^) is typical of agricultural soils (Table 1). The lower density in the C horizon with higher organic matter content was a result of buried stumps.

**Table 1:** Soil properties of different horizons near (8 m) the buffer strip.

Horizon	Units	Ap	B	C
Depth (m)	m	0–0.12	0.12–0.85	0.85+
Vertical Ks	m j^-1^	0.31	0.52	-
Horizontal Ks	m j^-1^	0.41	1.6	2.7
ρa	Mg m^-3^	1.40	1.43	1.36
P	m^3^ m^-3^	0.43	0.46	0.47
θv._-3 m_	m^3^ m^-3^	0.32	0.32	0.28
θv._-10 m_	m^3^ m^-3^	0.18	0.12	0.10
Sand	%	76.9	56.2	56.8
Silt	%	18.4	35.9	35.9
Clay	%	4.6	7.9	7.3
Organic matter	%	3.0	3.8	5.6
pH	—-	6.6	5.3	5.1

Ks: Saturated hydraulic conductivity

BD: Bluk density

P: Total porosity

WC_-3 m_: Volumetric water content at -3.0 m of matric potential

WC_-10 m_: Volumetric water content at -10.0 m of matric potential

B parameters are averages for the B_1_ and B_2_ horizons

Percent % is on a mass basis.

A mixture of indigeneous and invasive plants grows on the creek bank section of the buffer strip. The vegetation included *Calamagrostis canadensis* sp., *Phalaris arundinacea* L., *Agropyron repens* (L.) Beauv, *Elymus repens*, *Taraxacum officinale*, and others. During the growing season, vegetation along the creek bank was dense and homogeneous, growing to a height of about 1.4 m high, with a deep rooting system extending at least 0.6 m deep. The flat section of the buffer was also covered with grass, but vegetation did not grow as much in this area because of compaction. During spring 2011, the flat section was seeded with the ‘Cave-in-Rock’ variety of switchgrass (*Panicum virgatum* L.). Prior to seeding, this section was never worked but used only for machinery circulation.

**Fig 2:**
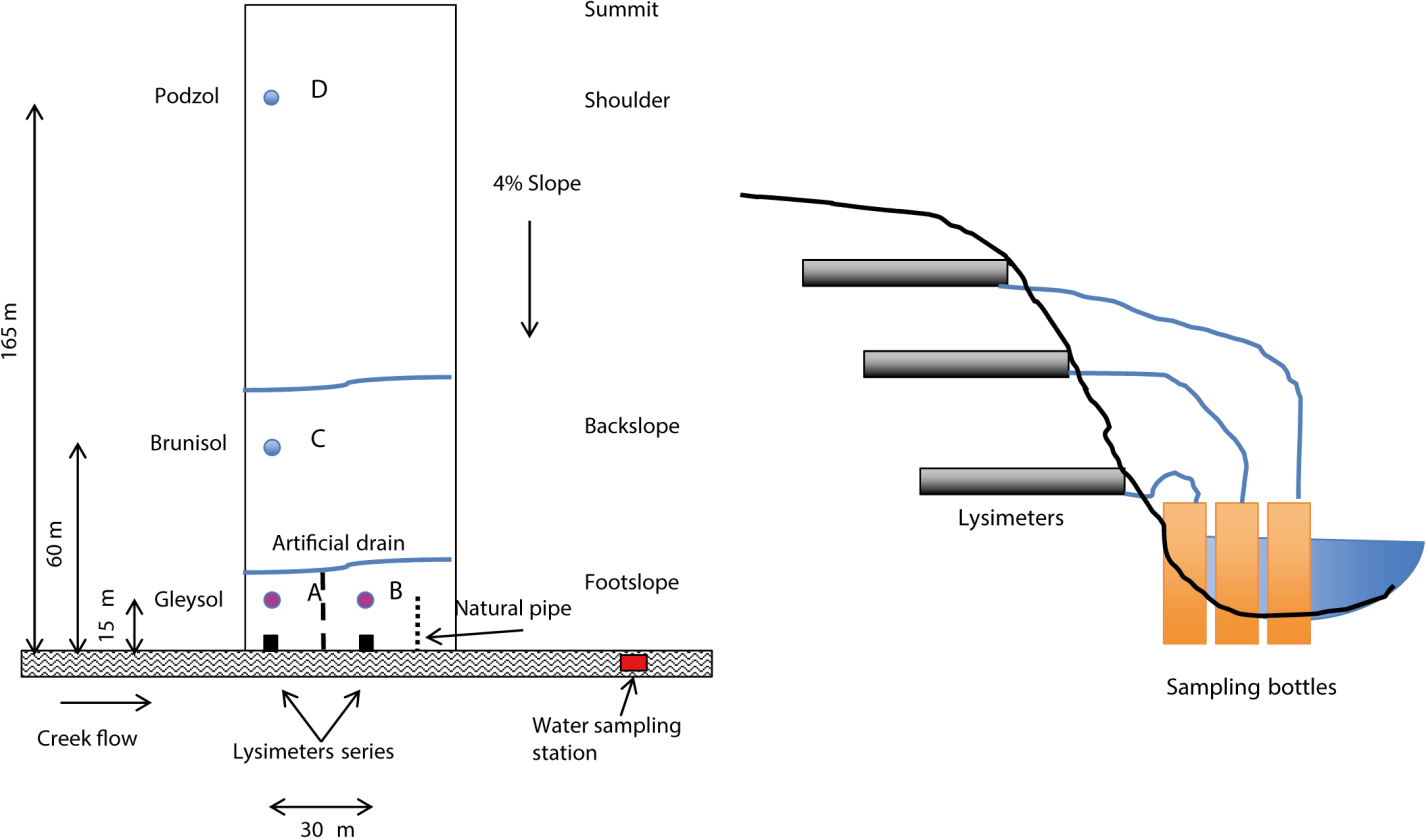
Schematic representation of the experimental set up and installation of the lysimeter plates (not scaled). The plots were not protected after tracer application, and the farm maintained its normal production routine. It is important to keep in mind, however, that the tracers were applied after fall soil tillage and the plots were sampled later that fall.

Four 2- x 2-m plots were established in the field. Three were aligned along distinct parts of the catena at 15 m (plot B), 60 m (plot C), and 165 m (plot D) from the creek (Fig 2). The fourth (plot A) was placed 15 m from the creek at the footslope, about 30 m away from plot B (Fig 2). Thus, A and B were along the creek at the footslope, while C was at the backslope and D was at the shoulder. Two tracers were homogeneously applied by hand at the soil surface using 20 L of water (Fig 1C). The tracer FD&C Blue #1 (Warner-Jenkinson Company) was applied at a rate of 125 g m^-2^ on all plots on 8 October 2010, while rhodamine WT (Sigma, San Diego, CA, USA) was applied at a rate of 2.5 g m^-2^ on the same day using the same solution and method, but only on plots A and B (close to the buffer strip). The tracers were chosen to represent weakly sorbed (Blue) and more sorbed (rhodamine) solutes. The Blue mimics phosphate [24] or atrazine [25], and rhodamine represents medium-sorbed pesticides [26] in low-sorbing soils. Tracer selection and application rates were based on detection limits, toxicity, and because they were used in previous researches [27–28]. They were never reapplied.

### Water, soil, and tracer sampling

Two series of zero-tension lysimeters were installed in the buffer strip (Fig 2) in May 2010. They were made of stainless steel and were 0.903 m wide, 0.506 m long, and 0.092 m high in the center. The lysimeters were aligned with plots A and B. One series was near (but not at) a visible lateral PF path, while the other series was installed where no PF was observed. They were installed at the interfaces between soil horizons at 0.12 (A and B_1_ interface), 0.24 (B_1_ and B_2_ interface), and 0.85 (B_2_ and C interface) m depth. One year passed before we started collecting water samples from the lysimeters in order to permit soil settlement. The lysimeter plates worked only when the soil surrounding the plates was saturated, since they only intercepted free water. They were positioned to only capture water moving horizontally or vertically in between two soil layers. The plates reacted independently of each other, since the activated ones were not always the same. When the creek rose too high, the deepest lysimeters were not sampled because the water level was higher than they were.

Lysimeter water collection occurred from May to September in 2011 and 2012 during intense rainfall events, *i*.*e*., when the creek rose to at least 0.25 m as detected by the water height detector in the creek. Tracer concentrations represent the average concentration recorded by the lysimeters over the 12 hrs during and after precipitation events. Collected water was transported through PVC piping and stored in Nalgene bottles until sampling. The samples were kept at 4°C in darkness until analysed.

Water was sampled in the creek about 60 m downstream from the lysimeter series. The autosampler collected 50 mL of water every 4 hours. These samples were combined into composite samples, each of which represented about 3.5 days of sampling. Additional samples were taken during heavy rainfall events: the autosampler collected samples hourly for 15 hrs whenever the creek reached a height of 0.25 m. In this case, the composite samples were made from 4 individual samples (average of 4 hrs). All samples were filtered and frozen before tracer analysis. Creek depth was measured daily in between rainfall events and hourly during precipitation. Water was also occasionally sampled from the outlet of a drain situated between both lysimeter series (Fig 2).

A meteorological station was installed on site to monitor daily air temperature, relative humidity, vapour pressure, wind speed and direction, direct and reflected solar radiation, and rainfall depth.

Trenches were dug very close to the lysimeters in November 2011 and May 2012 (2 profiles per date) for soil sampling in the buffer strip. Disturbed soil samples were extracted at a range of depths and distances from the creek. The samples were conserved at 4°C in darkness until tracer concentration analysis. Additional soil was sampled to characterize general soil attributes (Table 1) such as pH [29], texture [30], saturated hydraulic conductivity (Ks) with constant pressure head [31], water retention at -3.0 and -10.0 m of tension (WC_-3_ m, WC_-10 m_) [32], and water content. We also measured the bulk density (BD) [33] of the soil, from which we calculated total porosity (P) using the equation P = 1-BD/PD, assuming ρa, the particle density, to be 2.65 Mg m^-3^. Since the soils of the B_1_ and B_2_ horizons were very similar, they were often combined for measuring and interpreting their properties.

### Tracer analyses

Soil samples were dried for 24 hrs at 105°C prior to tracer extraction. A ratio 1:5 soil:water was used for tracer extraction [34]. Samples were shaken for 24 hrs and then centrifuged for 2 hrs at 4000 g (Beckman Coulter, Allegra 25 Re centrifuge). Water samples were also centrifuged prior to concentration measurement. The solutes were stored in darkness at 4°C until measurement.

A UV-visible spectrophotometer (model Genesys 6, Spectronic) set at 630 nm was used to measure Blue concentration [35–37]. The minimum and maximum detection limits for the Blue were 0.001 and 13 mg L^-1^. A luminescence spectrophotometer (model LS55, Perkin Elmer) was used to measure rhodamine concentration. The rhodamine was excited at 560 nm and emissions were measured at 577 nm [16]. The rhodamine detection limits were 0.08 and 25 x 10^−3^ mg L^-1^.

Tracer blanks (soil without tracers) were used to measure the natural noise for tracer analyses. Blanks received the same treatment as other samples. Noise levels for Blue and rhodamine were relatively low (Table 2) and were mainly due to organic matter and iron content. Blue was easy to extract with 67–79% efficiency (Table 2) because of its negative charge in acid soil and high water solubility (200 g L^-1^; http://www.jagson.com/food-color/brilliantblue.php, consulted on 4 November 2014). By contrast, less than 1∕3 of rhodamine was recovered (Table 2). Rhodamine losses were due in part to its stronger sorption on soil particles and greater sensitivity to light and heat, although the samples were protected from light and kept cold during storage. Extraction efficiency was very stable for the Blue, with a coefficient of variation (CV) < 5%, but was more variable for rhodamine (CV < 15%). Soil blanks (tracer without soil) were subjected to the same handling and storage procedures as the soil samples. Blue and rhodamine were lost at a rate of 0.08 and 4 g g^-1^ *100 month^-1^ of storage, respectively. Noise, extraction efficiency, and loss during storage and handling were used to correct concentration estimates.

**Table 2:** Background efficiency and linear sorption coefficient (Kd) of two tracers (FD&C Blue 1 and rhodamine WT) in different horizons near (8,) the buffer strip.

Horizon	Noise (absorbance and % of intensity)		Extraction efficiency (%)		Kd (cm^3^ g^-1^)		Kd ratio (unitless)
	Blue	Rhod.	Blue	Rhod.	Blue	Rhod.	Rhod./Blue
Ap	0.05	0.2	79	28	5.8	206	35.5
B	0.05	5	75	28	6.2	222	35.8
C	0.05	4.9	67	15	7.6	256	35.7

Percent % is on a mass basis

Parameters relative to B horizons are averages fro the B_1_ and B_2_ horizons.

Linear sorption isotherms (Kd) were calculated using a range of concentrations slightly larger than that found in the samples [34, 38]. The soil solution was gently shaken for 24 hrs to reach equilibrium, and extraction was then conducted using the method described earlier. The Blue Kd was about 35 times that of rhodamine (Table 2) and increased slightly with depth because of an increase in clay and organic matter content.

### Statistical analyses

Descriptive statistics were calculated using PROC UNIVARIATE in the SAS/STAT software v.9.1 (SAS Institute Inc., 2008). Data were transformed using the Box–Cox method to increase their normality when required [39]. Correlations were calculated using PROC CORR and PROC CANCORR, and regressions were calculated using PROC REG with repeated measures when applicable.

The tracer budget could not be estimated in this study since tracer concentrations were measured only during the unfrozen period, drains were rarely sampled, neither the concentration nor the flow in surface runoff were measured, and only a very small section of the buffer strip was monitored. In addition, tracer uptake by vegetation was not measured.

## Results and Discussion

### Precipitation and creek flow

The fall season of 2010 received more rain than the other seasons. Tracer application took place between two precipitation events in October, enabling the tracers to penetrate the soil surface before freeze-up. Some erosion occurred at the soil surface (Fig 1D) following an extremely strong precipitation event a few weeks after tracer application. During this event, gullies were formed and the grass of the buffer strip intercepted soil particles. Some of the Blue tracer (observed not measured) moved by runoff from the application plots to a location on the buffer strip distant from the lysimeters.

**Fig 3:**
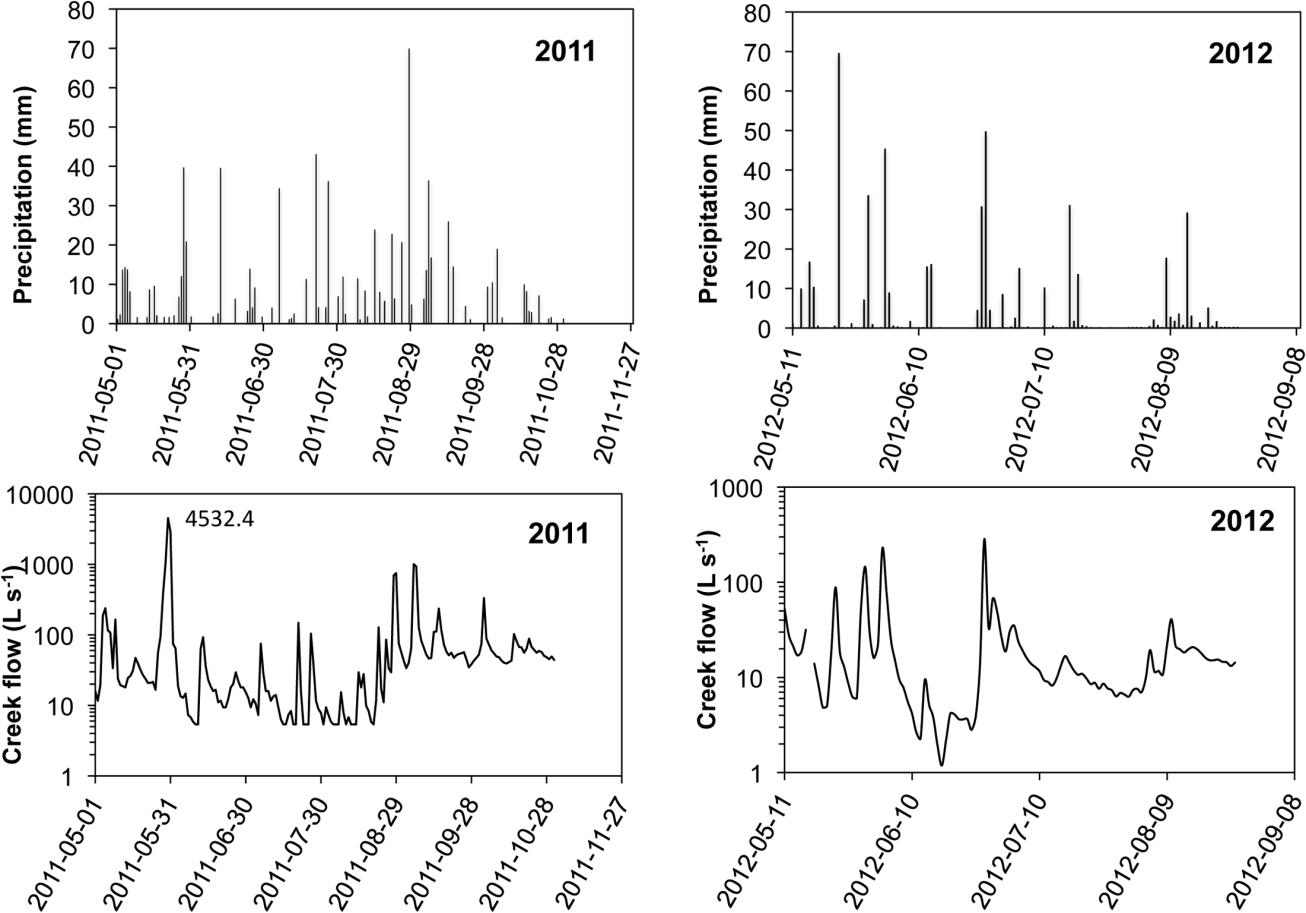
Precipitation and creek flow during 2011 and 2012.

The temperature (data not shown) and precipitation of winter 2011 were representative of the 30-year average. The month of May received one large (83 mm) rain event over a short period (Fig 3), representing about half of the 164 mm total for the month. Surface runoff and erosion were observed during this large rain event, and gullies developed throughout the field (Fig 1D). These gullies did not follow wheel tracks but were parallel to the slope. Some of the gullies reached more than 1 m wide. No gullies were observed in the measured area of the buffer strip. During this event, a portion of the sediment was trapped by the grass in the buffer strip, while another portion passed through the strip. This phenomenon seems to have occurred only during this extreme weather event and the prior one in fall 2010, although other intense rainfalls did occur during the summer (Fig 3). The mass of sediments that bypassed the buffer strip was not measured.

The months of May and June 2012 received more than 179 and 159 mm of rain, respectively, with 6 major precipitation events. Limited erosion but not gully formation was observed during these events. The other events of the summer were less important.

The creek flows year round (Table 3 and Fig 3), even during dry summers such as that observed in 2012. The minimum stream flow was 0.2 L s^-1^ in 2012. The maximum flows in 2011 and 2012 were 4 532 L s^-1^ (30 May 2011) and 3 776 L s^-1^ (30 September 2012). The average flow in 2011 was almost five times that of 2012 (Table 3).

**Table 3:** Descriptive statistics of creek height and flow during the studied period of 2012 to 2012 downstream from the lysimeter plates.

Parameter	Height (m)			Flow (L s^-1^)		
	2010	2011	2012	2010	2011	2012
n	261	224	20 738	261	224	106
Min	0.14	0.20	0.03	0.2	5.38	1.18
Max	0.65	1.11	1.03	3 776	4 532.3	283
Mean	0.24	0.28	0.19	40.7	96.4	21.82
STD	0.05	0.09	0.11	0.06	376.8	37.9

STD: Standard deviation

### Tracer concentration in the creek

The minimum Blue concentration in the creek was below detection limits (Table 4). Its maximum concentration in the creek was 0.15 mg L^-1^ in 2011 and 0.37 mg L^-1^ in 2012. The average Blue concentration in the creek was similar during both years (Table 4). Blue was detected half of the time in 2011, while it was detected only half as often in 2012.

**Table 4:** Descriptive statistics of tracer concentration (FD&C Blue 1 and rhodamine WT) in the creek, lysimeter plates, drain, and buffer strip soil over the 2-year period (fall 2010 to fall 2011 and fall 2011 to fall 2012).

Position	Parameter	2011		2012	
		Blue (mg L^-1^)	Rhodamine (10^−3^ mg L^-1^)	Blue (mg L^-1^)	Rhodamine (10^−3^ mg L^-1^)
Creek	n	50	50	33	33
	Min	ND	0.23	ND	0.29
	Max	0.15	13.6	0.37	3.47
	Mean	0.02	0.93	0.03	0.91
	Std	0.03	2.04	0.08	0.83
Lysimeters	n	38	22	14	14
	Min	ND	0.10	ND	0.42
	Max	50.0	4.05	0.44	2.60
	Mean	3.07	1.29	0.08	1.40
	Std	11.1	1.05	0.11	0.69
Drain	n	0	0	20	20
	Min	NA	NA	0.001	0.12
	Max	NA	NA	0.017	0.47
	Mean	NA	NA	0.002	0.26
	Std	NA	NA	0.004	0.08
Soil	n	23	23	49	49
	Min	ND	2.07	ND	4.33
	Max	2.18	38.1	3.46	113.1
	Mean	0.46	16.5	0.74	29.3
	Std	0.77	10.4	0.95	19.2

ND: Not detected; NA: Not available; STD: Standard deviation.

**Fig 4:**
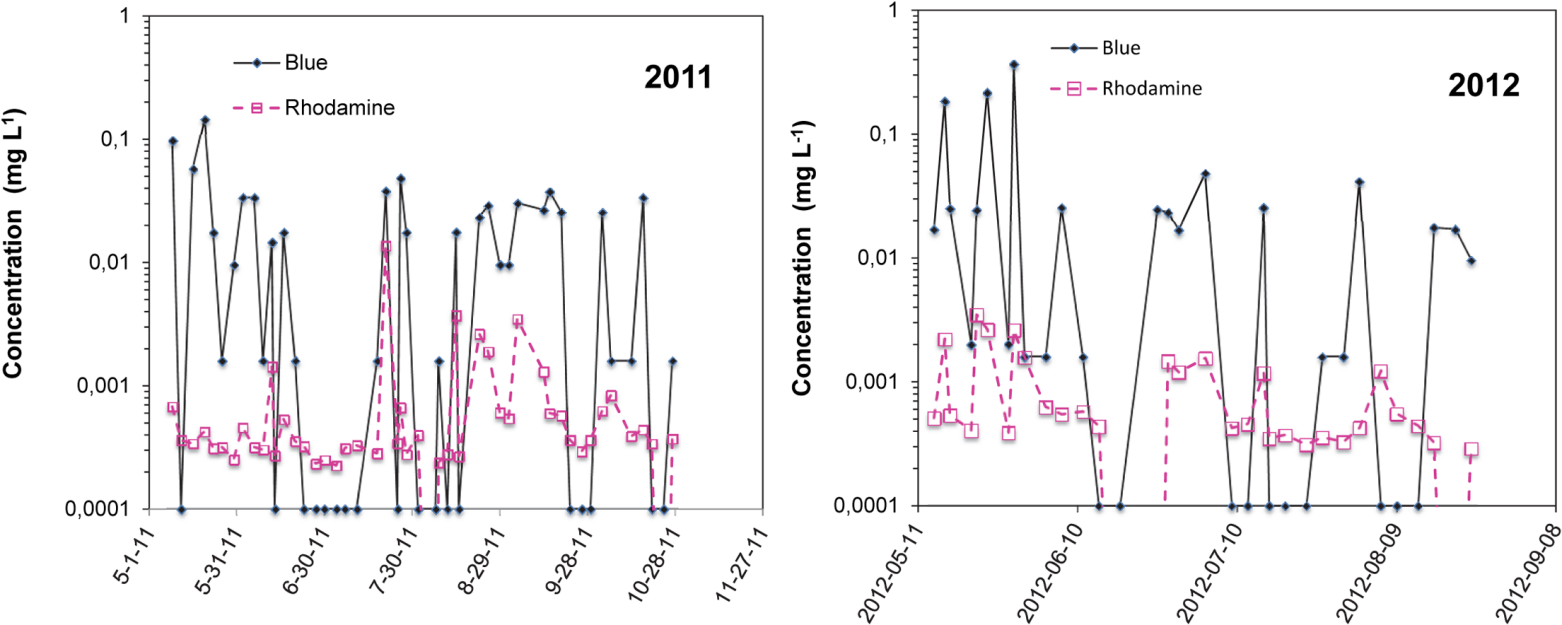
Blue and rhodamine concentration in the creek in 2011 and 2012.

Mean rhodamine concentration in the creek was similar during both years, but its concentration peaks were higher in 2011 than in 2012 (Table 4). Maximum rhodamine concentration reached 13.6 x 10^−3^ mg L^-1^ and 3.47 x 10^−3^ mg L^-1^ during rain events in 2011 and 2012 (Table 4). The maxima were respectively 13 and 3 times higher than the mean concentrations in 2011 and 2012. The tracer was almost always detected in the creek, being found as frequently in 2011 as in 2012 (Fig 4). When rhodamine was detected in high concentrations, Blue was also detected, but its concentration did not follow that of rhodamine.

In neither case, tracer concentration was significantly correlated to creek flow (data not shown). Neither time lag, actual concentration, relative concentration, nor mass flow (creek flow x tracer concentration) improved these correlations. Rhodamine was almost always present in the background, but its concentration did not necessary increase with creek flow.

Rather than being correlated to creek flow, tracer concentrations were better correlated, although weakly, to the cumulative amount of precipitation between concentration measurements. The Blue concentration tended to increase (linear regression, P = 0.05, R^2^ = 0.17) with the total precipitation between two measurement events. This may be due in part to spatial and temporal field variability in precipitation. Precipitation amounts greatly varied over very short distances at this site, changing spatial dilution from one rain to another. In addition, tracers already in the creek bank may have been released when the water level rose.

Tracer concentrations in the creek were not significantly correlated with one another during 2011, but were during 2012. Rhodamine concentration tended to increase with Blue in 2012 (linear regression, P = 0.05, R^2^ = 0.35). Blue concentration ranged across almost three orders of magnitude, while rhodamine ranged across only two.

### Tracer concentration in the drain

Blue appeared in the drain just 5 days after its application. The drain was situated several meters away from the application plots (the closest plot was 12 m away). No surface runoff containing Blue was apparent at that time, but we could visually detect it in the drain. This happened two weeks before the heavy rainfall event that led to gully formation and important surface runoff. Therefore, very fast tracer movement occurred belowground to reach the drain, with movement in part perpendicular to the surface slope. Since rhodamine could not be observed as easily with the naked eye, we do not know if its movement was associated with surface runoff.

Drain water samples contained rhodamine most of the time, but Blue was more rarely present. When Blue was detected, its concentration was strongly correlated to that of rhodamine (linear regression, P = 0.01, R^2^ = 0.87).

### Tracer concentration in lysimeter plates

**Fig 5:**
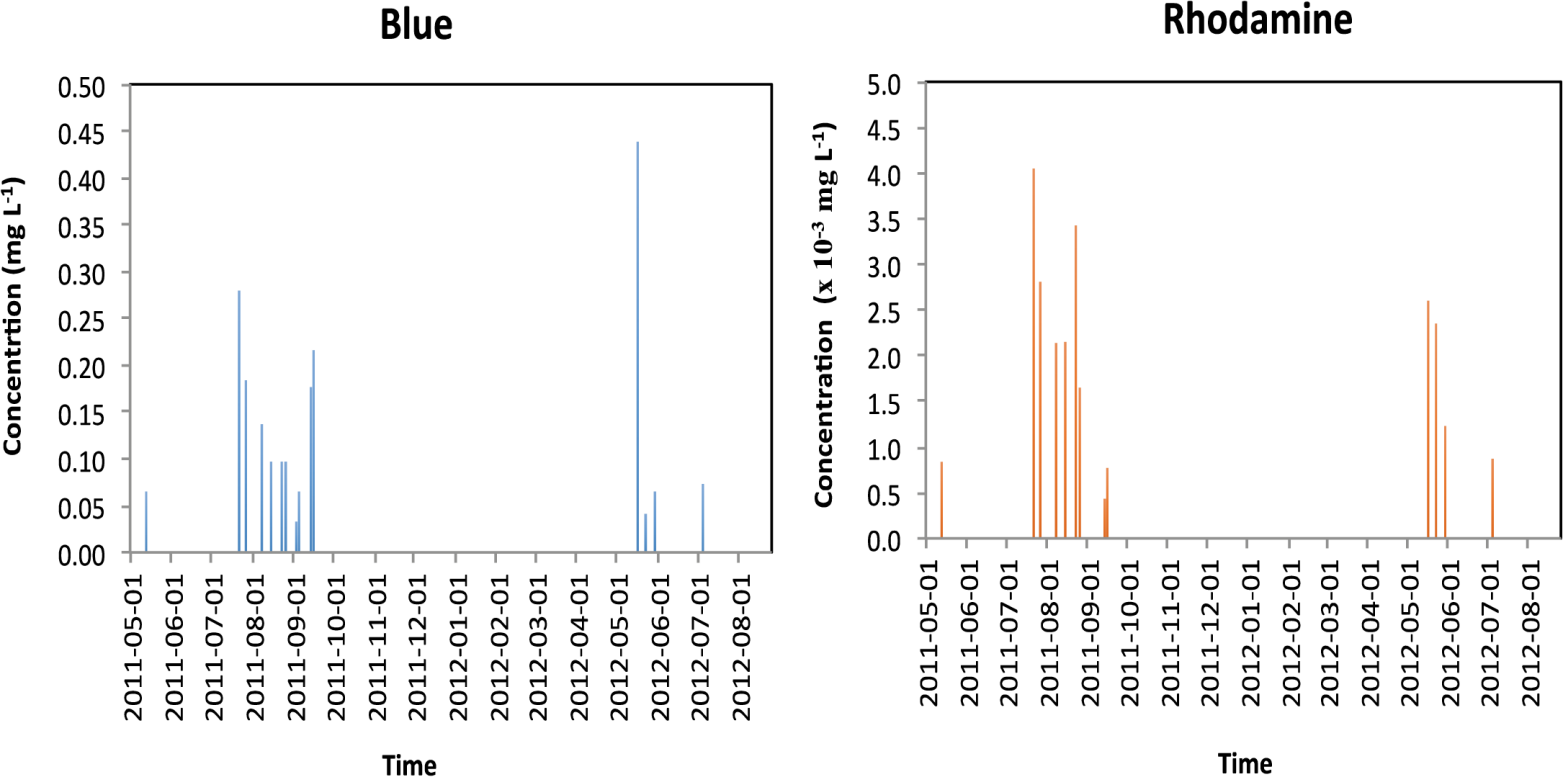
Maximal Blue and rhodamine concentrations in the lysimeter plates during both years (no samples were taken from November to beginning of May).

The lysimeters rarely worked, except during intense or long precipitation events, resulting in scarce samples. These samples showed average Blue and rhodamine concentrations in the same order of magnitude as those in the creek during rain events (Table 4). However, the maximum concentrations of Blue (50.0 mg L^-1^) and rhodamine (4.05 x 10^−3^ mg L^-1^) were higher than those in the creek in 2011 (0.15 mg L^-1^ for the Blue and 0.37 x 10^−3^ mg L^-1^ for the rhodamine) and lower in 2012 (0.44 mg L^-1^ for the Blue and 2.60 x 10^−3^ mg L^-1^ for the rhodamine). There was always at least one of the tracers in the water in at least one lysimeter. Rhodamine was more frequently observed in lysimeters (in several lysimeters at the same time) than Blue, which was detected almost exclusively in the deepest lysimeters. When Blue was detected, its concentration was at most ten times higher than that of the rhodamine (Fig 5).

No significant regressions or correlations were found between creek and lysimeter concentrations, with or without time lag analyses, for either tracer at any depth (data not shown). The only significant (P = 0.05) correlation found between lysimeter concentrations and other parameters concerned Blue concentration in the deepest lysimeter, which was weakly correlated (linear regression, P = 0.05, R^2^ = 0.18) with precipitation, although only in 2011. These results indicate that Blue was not carried into the buffer strip by water from the creek, but rather from the upper part of the field through deep percolation [40].

### Tracer concentration in soil

**Fig 6:**
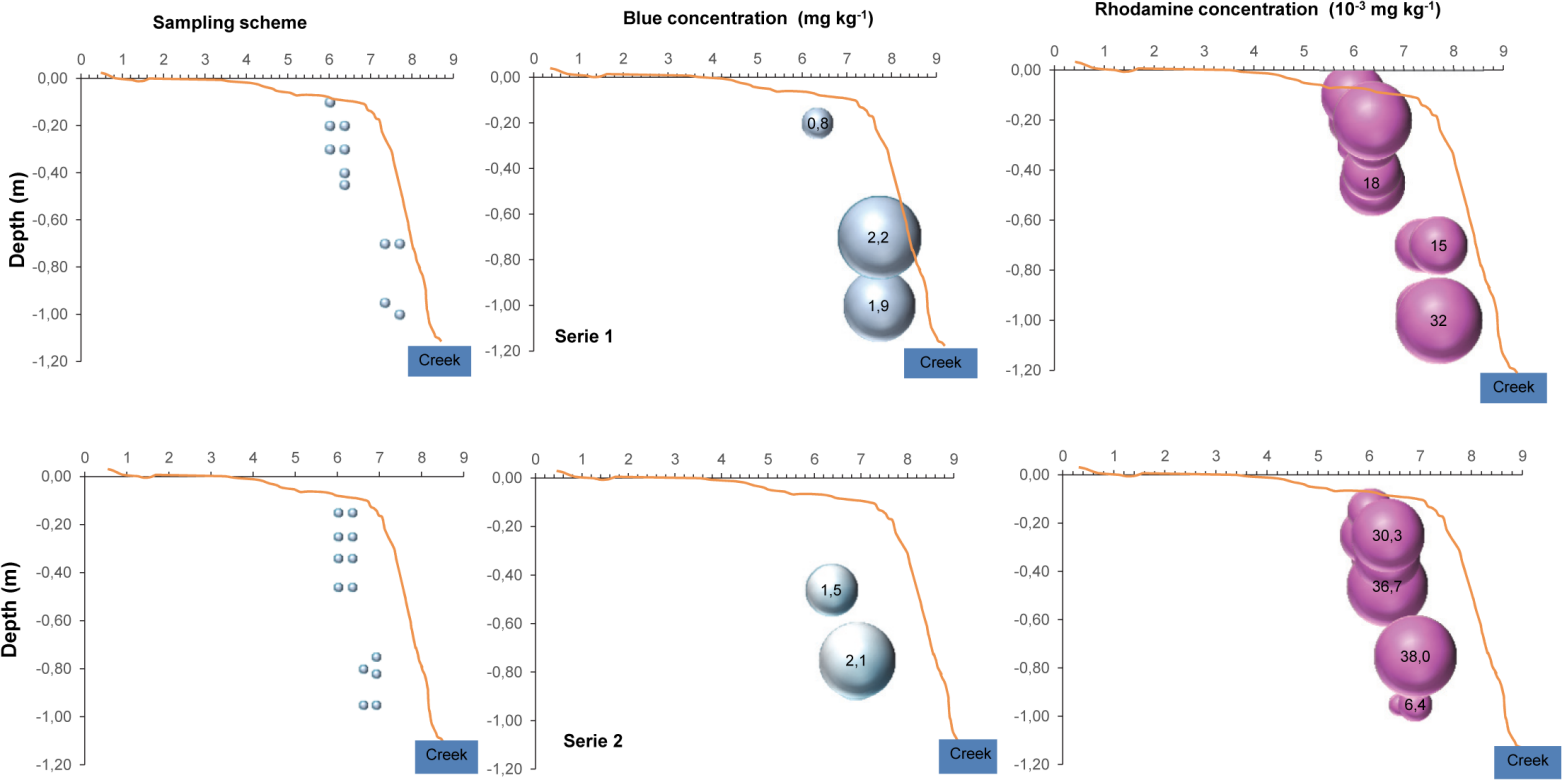
Sampling scheme and distribution of Blue and rhodamine in two series of measurements in the buffer strip soil in November 2011. The absence of Blue circles at spots corresponding to the sampling scheme indicates no Blue detection. Circle size indicates tracer concentration.

**Fig 7:**
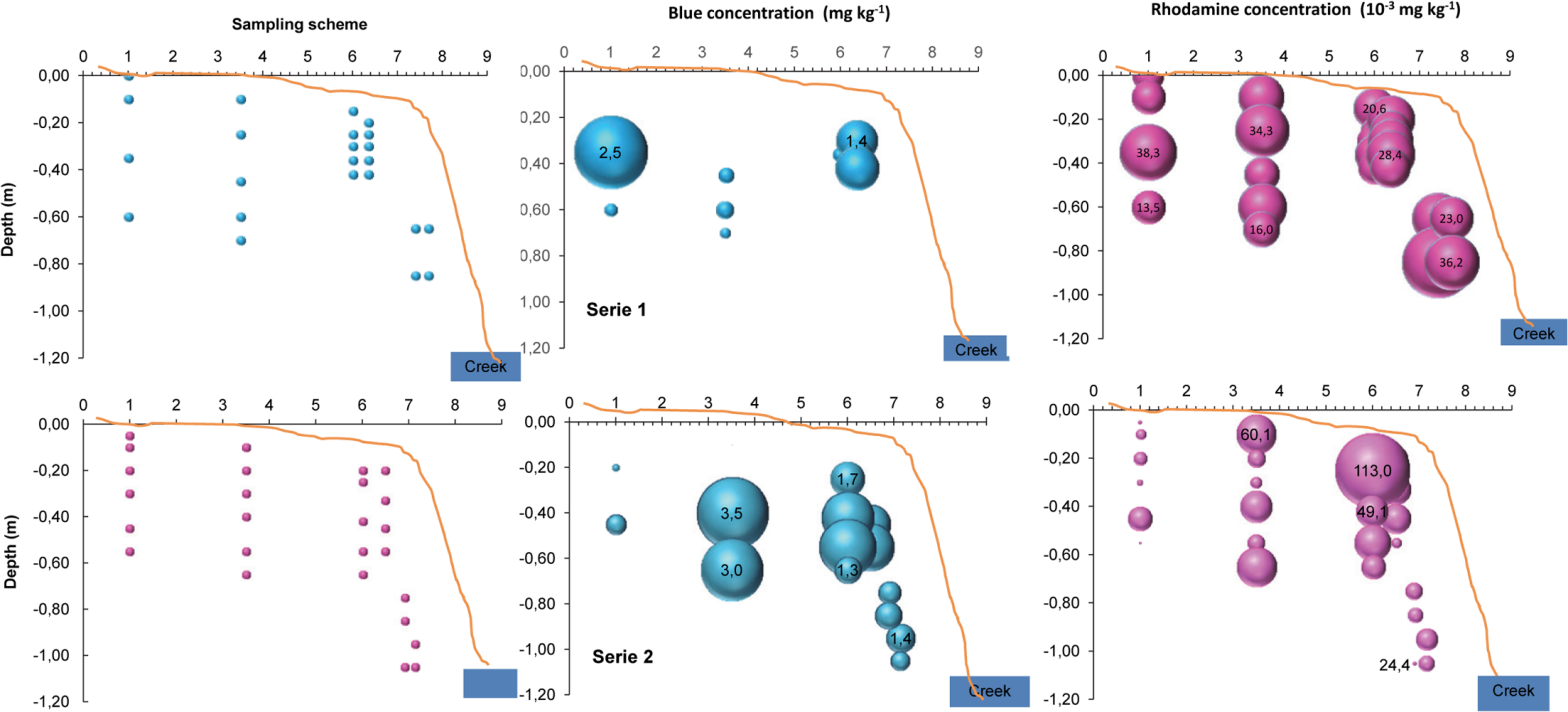
Sampling scheme and distribution of Blue and rhodamine in two series of measurements in the buffer strip soil in May 2012. The absence of Blue circles at spots corresponding to the sampling scheme indicates no Blue detection. Circle size indicates tracer concentration.

The tracers were already present deep in the buffer strip a year after their application (Fig 6), and were even more concentrated six months later (Fig 7). The tracers reached at least 0.8 m depth in the middle of the buffer strip and more than 1.2 m depth close to the creek 6 months after their application.

Tracer concentration in the buffer strip soil showed important spatial variability. In general, the variability in Blue distribution was higher than that of rhodamine, with spots of very low and very high concentrations. The Blue was more concentrated at depth than near the soil surface, where it was not detected. By contrast, the rhodamine was more homogeneously distributed with depth and between sampling events (Figs 6 and 7). Since Blue is stable under natural light and very stable under any soil conditions (very slowly degraded) while rhodamine is sensitive to light and is consumed by soil microorganisms, the absence of Blue at the soil surface probably indicates that it did not move to the measured locations at the soil surface, but rather penetrated the buffer strip by underground lateral movement from upslope [22, 41]. An alternative interpretation might be that Blue was not found at the soil surface because it was flushed away during runoff events, since it is weakly sorbed on soil particles and highly soluble. However, if that were the case, we would have observed Blue with the naked eye at the soil surface at other times, since we could detect it belowground. In addition, we would expect a stronger relationship between rainfall depth and Blue concentration in the creek.

### Additional observations

The standard deviation of Blue was higher than that of rhodamine in all four compartments (creek, lysimeters, soil, and drain) (Table 4). The highest maximum and mean for Blue occurred in lysimeters in 2011, while in 2012 it occurred in the buffer strip soil (Table 4). By contrast, the highest maximum concentration values and highest means of rhodamine were observed in the buffer strip soil during both years (Table 4).

We observed strong resurgences of water along the buffer strip at very specific narrow points. Some of these points corresponded to holes (also called natural pipes) associated with old, decayed stumps, some of which were large enough to accommodate the hand of an adult person. These resurgence points in the buffer strip, visible with the naked eye, flowed directly into the creek and were active mainly during rainfall events, but also between them. Any PF pathways that passed through the application plots are likely to have transported tracers at higher concentration. Otherwise, such paths would dilute the tracers. Thus, depending upon the paths during each rainfall event, these pipes may have dilute or carried more tracers through the buffer strip. One of these resurgence points was located close to a lysimeter series and the trench used to measured tracer concentration in the buffer strip soil but did not pass through it, but not directly on the measurement points. We therefore did not measure the direct effect of these resurgence points on tracer transport through the buffer strip.

Another narrow resurgence was identified at the footslope slightly uphill from plots A and B. Blue was present at this location, visible with the naked eye. This resurgence point seems to be stable over time as it rewetted after rainfalls and more Blue appeared as time went on. No other such resurgence was observed in the field. Because of its uphill position compared to plots A and B, the Blue could not come from these plots but must have come from higher in the catena (plots C and/or D) and this, through underground preferential lateral flow (LF) since its appearance was very localised.

In addition, scarce, localised, narrow points of seeping were observed when trenches were dug in the buffer strip for soil sampling. Some of the samples also contained the Blue in concentration high enough to be visible with the naked eye.

### Movement through advection–dispersion of solutes in the soil matrix

The generally accepted way to describe solute transport in soil is the standard advection–dispersion equation [42], which can be written as follows:

$$R\;dC_{l}/dt=De\;d^{2}C_{l}/dz^{2}-V\left(dC_{l}/dz\right)-rs$$ (1)

R is the retardation factor (unitless), *C*_*l*_ is the liquid phase solute concentration (mg L^-1^), t is the time (day), *D*_*e*_ is the effective coefficient of dispersion (day^-1^), *z* is the distance (m), *V* is the water velocity (m day^-1^), and rs is the solute appearance or disappearance. In this case, the tracers are the solute.

The shortest time required for the tracer to reach the creek from the application plots when moving by the standard advection–dispersion process, *i*.*e*., by matrix flow, occurs during saturated conditions. If diffusion, appearance, and disappearance are assumed to be negligible compared to the hydrodynamic dispersion (in order to calculate the fastest possible transport time and simplify the equation), the time (Time) to reach the creek can be estimated as follows [38]:

Time=LR/V (2)

$$R=1+\left(BDKd/WC_{v}\right)\;\text{and}\;V=q/{\text{WC}}_{v}=q/P$$ (3)

θ _v_ is the volumetric soil water content (m^3^ m^-3^). WC_v_ = P at saturation. The q is the flux (m s^-1^). Given the distance from the application plots to the creek (L = 15 m), the water flux is:

q=−KsdH/dz (4)

dH/dz is the hydraulic gradient and equal to 1.0 under saturated conditions.

Using these equations, the time for tracers to reach the creek from the application plots is easily estimated from the P, Kd, Ks, and R values given in Tables 1, 2 and 5. The fastest time to reach the creek was calculated for each horizon assuming lateral movement. The time required to vertically cross each horizon was added to time for covering the lateral distance to evaluate the time to move from the applied plot to the creek (Table 5).

**Table 5:** Estimation of the shortest time required for water and tracers to reach the B horizon in the middle of the buffer strip from the closest application plots (Plots A and B) assuming constant saturated conditions and movement exclusively in the matrix by hydrodynamic dispersion.

			To reach the B horizon of the strip		To reach the creek	
Tracer	Horizon in the applied plots	R (unitless)	Water (days)	Tracer (years)	Water (days)	Tracer (years)
Blue	Ap	19.9	10.4	0.57	15.6	0.85
	B	20.6	2.5	0.14	3.8	0.21
	C	23.0	1.7	0.11	2.6	0.17
Rhod.	Ap	671	10.4	19.3	15.6	29
	B	644	2.5	4.8	3.8	7.2
	C	649	1.7	3.5	2.6	5.3

R: Retardation factor = 1+(ρD Kd/ θ K_v_); at saturation WC_v_ = P; Time = (LR)/v, v = q/ θ _v_), L = 15 m (distance from the creek); q = -Ks dH/dz, dH/dz: Hydraulic gradient = 1 under saturated conditions.

Molecular diffusion is assumed negligible compared to the hydrodynamic dispersion.

The B parameters are averages for the B_1_ and B_2_ horizons

The predicted values suggest that the Blue could have reached the B horizon of the buffer strip within a few months while rhodamine should not have reached the buffer strip before the third year (Table 5). Comparatively, tracer concentrations in the buffer strip soil and in the lysimeters indicated that both tracers were present in the subsurface as soon as a year after their application.

According to the predictions, it would take between 2.6 and 15 days, depending upon the horizon, for the water to reach the creek (Table 5), comparatively to 61 days and about 10 months for the Blue and 5 to 29 years for the rhodamine to cover the same distance under the same conditions (Table 5). In fact, both Blue and rhodamine moved much faster than suggested by the predictions (about 12 times for Blue and 390 times for rhodamine), since they appeared in the creek only 5 days after their application. Actually, both tracers moved almost as fast as the water. Considering that the soil was not always at saturation, which reduces water movement, the difference between the predicted and the observations was even greater. In addition, the differences between the predicted and the observed times were far more important for rhodamine than for Blue. These results suggest that the actual tracer movement occurred not only through matrix flow by advection–dispersion, but also through faster processes such as subsurface PF.

Since the movement of both tracers was comparable to those of water and they were applied at the same time, position, and depth, we can use their Kd or R ratios to evaluate whether the flows occurred through the soil matrix or PF. If the ratio of the time required to cover a certain distance (slower tracer ∕ faster one) is equal to their Kd or R ratios, then the movement occurred by advection–dispersion within the soil matrix (matrix flow). If the ratio of the time they took to cover a distance is smaller than their Kd or R ratios, then they move not only through the soil matrix but also through PF paths. If the ratio of their time tends toward 1.0, *i*.*e*., they moved at about the same speed, then they moved almost exclusively by PF.

The Kd and R ratios of rhodamine/Blue are about 35 (Tables 2 and 5). Both tracers appeared in the creek and in the drain 5 days after their application, without apparent surface transport (R/R = 1). In addition, they appeared in the buffer strip in a ratio of at most 2. Therefore, important PF occurred early after their application and thereafter.

### Combining observations and tracer properties

#### Occurrence of several PF processes

Considering that (1) the Blue would take at least a couple of months and rhodamine more than 5 years to reach the creek under matrix flow at saturation; (2) it would take at least 35 times longer for rhodamine than Blue to reach the creek with advection-dispersion transport; (2) Blue and rhodamine were observed in the creek only a few days after their application in the field; (3) pipe flows were observed along the buffer strip; (4) a narrow point of resurgence containing the Blue occurred at the footslope; (5) narrow points of seeping containing the tracers were observed within the buffer strip soil; and (6) drain flows were observed to transport the tracers both early in the experiment and thereafter, then multiple belowground PF processes have occurred in the field.

In addition, (1) the soil changes along the catena; (2) the Blue was applied on all 4 plots rather than close to the creek as for rhodamine; (3) the Blue concentration was null at the soil surface but significant in the subsurface; (4) the Blue concentration deep in the buffer strip was maintained over time; and (5) its concentration increased at specific downhill at narrow spots over time. Thus, we can conclude that, among different belowground PF processes, lateral PF from uphill was important.

Because Blue concentration was not strongly correlated to precipitation, the active PF paths may not always have been the same. Some PF paths may be activated very rapidly, but may require specific conditions to be activated [41]. These may have been responsible for the early arrival of the tracers in the creek. It is also often the case that PF paths cover only a short distance, and connections between PF paths require matrix flow paths. Connected matrix paths require more rain and take more time to be activated. The transport network is thus enhanced with precipitation duration by a combination of PF and matrix flow paths [43]. When rain stops, the connectivity between paths is broken because the preferential flows quickly stop. Variations in the initial soil conditions (*e*.*g*., water distribution), intensity and duration of precipitation events, co-existing types of PF processes, and remaining active flow paths change the active network from one rain to another. Thus, the occurrence of different PF processes, the fact that they did not take place at the same time or at the same place during every rainfall in addition to the fact the PF paths interact with one another enhance the spatial and temporal variability of tracer movement [44]. It makes it difficult to establish correlations between precipitation events, creek flow, and tracer concentrations in different compartments (creek, drain, lysimeter, soil).

#### Differences in tracer movement

Blue concentration in the different compartments (creek, drain, lysimeter, soil) showed higher spatial and temporal variability than that of rhodamine. Rhodamine was consistently measured in the creek, in the lysimeters, and in the drain, while the presence of Blue was more intermittent. Peaks of Blue and rhodamine concentration did not occur at the same time. In addition, rhodamine was present at the soil surface, despite being sensitive to light and degradation processes. By contrast, the Blue was observed at the soil surface only at the resurgence spot and directly at the application spots. Otherwise, it was observed only in the subsurface.

These results are counterintuitive, since Blue was applied at the same time as rhodamine, at a higher concentration, and on four rather than on two plots. It is also less sensitive to degradation. We would thus expect the Blue to be observed in the different compartments more frequently than rhodamine. If these observations were due to the detection limits of our apparatus, we would expect high rhodamine concentrations to be detected whenever high Blue concentrations were detected, since the Blue detection limit is 100 times higher than that of rhodamine (*i*.*e*., less precise). This was not often to the case. The difference in temporal variation between both tracers was therefore not due to detection limits.

The higher variability of Blue concentrations was due in part to its higher solubility, decreasing its response time to rainfall events. Rhodamine moved not only in the subsurface, but probably also at the soil surface in runoff because of its higher sorption on soil particles. The distribution of the two tracers differed at the footslope, with both being present at some spots (the application plots) but only Blue being present at some spots (caused by lateral flow from uphill) and only rhodamine being present at others (*e*.*g*., soil surface). In addition, it is known that PF paths that crossed a location where a tracer was present carried the tracer further downhill, while those that did not pass through a tracer location diluted the tracers along their way [45]. Therefore, the two tracers used a variety of differing paths to move through the field [40].

#### Buffer strip efficiency

The existence of PF from uphill, the quick response of tracer movement to precipitation, the increasing concentration of Blue downhill, and the differing behaviour of both tracers indicate that all parts of the catena were involved in solute transport to the creek. Both Blue and rhodamine moved through lateral PF in the subsurface and reached the creek in part by paths below the buffer strip, bypassing it. Therefore, the buffer strip design did not provide for the effective interception of soluble components in the presence of subsurface PF in the field.

The fact that the tracers were detected in the buffer strip suggests that it does have some ability to intercept solutes [6]. But a significant portion of the tracers bypassed the buffer strip, as shown by tracer concentration in the creek. This happened through underground flow paths such as PF. Even those solutes that are intercepted by the buffer strip may be released at later times to the creek. Indeed, lysimeter and creek concentrations indicated the release of both tracers from the buffer strip during rain events.

In sum, several mechanisms were found to reduce the ability of the buffer strip to intercept solutes. (1) The tracers passed beneath the buffer strip after having reached it partly through PF paths throughout the field. (2) The tracers bypassed the buffer strip along PF paths, such as pipe flows, within the buffer strip itself, decreasing its underground efficiency. In addition, gullies occurred at the soil surface in the field and in the buffer strip, bypassing it and decreasing its efficiency at the soil surface (however, we did not measure tracer transport by gullies). (3) Another portion of the tracers moved through drains, further decreasing the efficiency of the buffer strip once the soluble tracers had infiltrated into the soil from different points in the field. Thus, drains, pipe flow, gullies, and lateral PF, which occurred throughout the catena, all decreased the effectiveness of the buffer strip, particularly in the case of more retarded solutes (e.g. rhodamine comparatively to Blue).

### Implications

Many governments encourage farmers to establish buffer strips in order to decrease the transport of contaminants such as nitrate, phosphorus, and pesticides toward surface water. Based on the combined criteria of the FIHOQ [46], the MDDELCC [47], and the review conducted by Dorioz *et al*. [4], the buffer strip examined in this study should have been effective at intercepting contaminants since it is more than 7 m wide, it is densely vegetated, it is covered by herbaceous species over its entire surface, it features well-rooted plants along most of the creek bank, its soil is untilled, and no soil amendments have been applied to it. However, our results clearly show that this buffer strip cannot effectively filter surface-applied soluble contaminants: subsurface flows such as PF carry these solutes, even sorbed ones, downward and then laterally underneath, through, and over the buffer strip. The fact that PF occurs in most of the soils around the world [41] suggests that this could be a problem for buffer strips in many locations. It may also explain why agricultural contaminants are still so often present in Quebec rivers despite the widespread use of buffer strips in the province [21, 47].

In addition to the above new information, we know that problems can occur after buffer strips accumulate contaminants [4]. When the soil reaches its maximum sorption capacity, it may then release these contaminants [18], particularly through PF paths such as those observed belowground in the buffer strip in this study. It may then become a source rather than a sink of contaminants [7].

It is important to consider how the design of buffer strips might be modified [48] to improve their ability to capture soluble contaminants, particularly in the subsurface. Buffer strips could be constructed with filtering materials added belowground. Such filters could contain a highly sorbing material that would be removed on a regular basis and used elsewhere where nutrients are needed. Considering the cost and practical difficulties of this approach, however, the use of such filters might be limited just to hot spots, *e*.*g*., where subsurface bypass (PF) controls most of the water movement toward surface water.

It has also been suggested that the addition of perennial plants with deep root systems would be an efficient way to extract some proportion of contaminants in the subsurface [4]. But large, deep roots favour PF [49]. It would thus be preferable to use plants with fine, deep, fast-growing root systems that can tolerate a high water table. Such plants could be expected to remove some contaminants, but they would not be likely to have much effect on contaminants moving very rapidly by PF. To prevent contaminants from returning to the surface of the buffer strip and surface water once extracted by plants, partial harvesting of the plants, as suggested by Dorioz *et al*. [4] and Roberts *et al*. [11], might also be important. However, soil disturbance should be minimized during such harvests because disturbances would favour soil detachment and the development of further surface PF paths, such as gullies.

We were able to detect the presence of Blue in the creek with the naked eye with an applied mass of 125 g m^-2^, the equivalent of 5 x 10^−5^ kg ha^-1^, within just a couple of weeks after its application. Such concentrations in the creek would be considered dangerously high in the case of highly toxic chemicals, such as certain pesticides, and for contaminants that, even at small concentrations, affect the environment (*e*.*g*., antibiotics). We thus expect these observations to be true for soluble contaminants applied at high concentrations, such as nitrates, but also for highly toxic contaminants applied at low concentrations.

## Conclusion

The concentration of two soluble sorbed tracers was measured in a creek, in lysimeter plates installed in the buffer strip, and in the soil of a riparian buffer strip naturally vegetated with grass during a two-year period. These data and other field observations indicated the existence of important preferential flows (PF) in the field and tracer movement both beneath the buffer strip and through it via lateral PF, drains, and pipe flow. A proportion of both tracers may have used the same pathways to reach the creek, while another portion used different pathways. The Blue movement demonstrated that the entire catena was involved in the long-range transport of this tracer toward surface water, through both very fast and slow paths, although field slope was only 4%. Consequently, although the buffer strip was managed according to recommended methods the transport occurred through subsurface lateral PF processes, reducing the ability of the buffer strip to intercept contaminants. Buffer strips may be upgraded either by the addition of filtering agents installed in the subsurface at hot spots, which could be replaced at a specific interval or by the use of plants with deep, fine root systems to take up the contaminants, after which they could be removed by harvesting the plants.
